# DeepPredict: a state-of-the-art web server for protein secondary structure and relative solvent accessibility prediction

**DOI:** 10.3389/fbinf.2025.1607402

**Published:** 2025-06-06

**Authors:** Wafa Alanazi, Di Meng, Gianluca Pollastri

**Affiliations:** ^1^ School of Computer Science, University College Dublin, Dublin, Ireland; ^2^ Department of Computer Science, College of Science, Northern Border University, Arar, Saudi Arabia

**Keywords:** protein structure prediction, web server, deep learning, bioinformatics, protein language models, computational biology

## Abstract

DeepPredict is a freely accessible web server that integrates Porter6 and PaleAle6, two state-of-the-art deep learning models designed for protein secondary structure prediction (PSSP) and relative solvent accessibility (RSA) prediction, respectively. Built on an advanced deep learning framework, DeepPredict leverages pre-trained protein language models (PLMs), specifically ESM-2, to eliminate the need for multiple sequence alignments (MSAs), enabling rapid and accurate predictions. Compared to existing methods, DeepPredict outperforms in both PSSP and RSA prediction tasks, delivering state-of-the-art performance. The server offers a user-friendly interface, catering to both computational biologists and experimental researchers. DeepPredict is available at [ https://pcrgwd.ucd.ie/wafa/] with comprehensive online documentation and downloadable example datasets.

## 1 Introduction

Accurately predicting protein secondary structure and relative solvent accessibility is essential for understanding protein function, stability, and interactions. Traditional methods rely on evolutionary information derived from multiple sequence alignments (MSAs), which can be computationally expensive and ineffective for proteins with limited homologous sequences. Recent advancements in deep learning and protein language models (PLMs) have enabled the development of highly accurate, MSA-free predictors that significantly enhance computational efficiency while maintaining high predictive accuracy (Ismi et al., 2022).

DeepPredict integrates two state-of-the-art tools to advance protein structural analysis. It employs Porter6 (Alanazi et al., 2025a) for secondary structure prediction, offering both three-state (SS3) and eight-state (SS8) classifications. Additionally, it incorporates PaleAle6 (Alanazi and Meng, 2025) for relative solvent accessibility (RSA) prediction, supporting binary classification (RSA_2C), four-class classification (RSA_4C), and real-valued RSA predictions. By combining these powerful models, DeepPredict delivers comprehensive insights into protein secondary structure and solvent accessibility.

Designed as a unified platform, DeepPredict provides a seamless solution for protein structural analysis, offering both secondary structure and solvent accessibility predictions in a single tool. The DeepPredict web server builds on these advancements, delivering a freely accessible, user-friendly interface for researchers at all levels. It enables rapid and accurate predictions for both large-scale datasets and individual protein sequences, catering to diverse research needs in structural bioinformatics.

While DeepPredict focuses on one-dimensional protein structural properties—secondary structure and solvent accessibility—complementary tools such as AlphaFold and ESMFold predict full 3D protein structures. These 3D predictors are typically computationally intensive; AlphaFold, for example, relies on alignment-derived features and structural templates (Bertoline et al., 2023; Alanazi et al., 2025b). In contrast, DeepPredict provides rapid and lightweight per-residue structural insights, making it well-suited for high-throughput screening and for use as input features in downstream 3D modeling or protein function prediction pipelines.

This manuscript presents the design, implementation, and performance of the DeepPredict web server while exploring its potential applications in computational biology and structural bioinformatics. The DeepPredict web server is freely accessible at [https://pcrgwd.ucd.ie/wafa/].

## 2 Materials and methods

### 2.1 Overview of prediction algorithm

DeepPredict leverages a deep learning framework optimized for protein secondary structure and relative solvent accessibility (RSA) prediction. At its core, the framework integrates a hybrid convolutional bidirectional recurrent neural networks (CBRNN) architecture, which synergistically combines the strengths of convolutional neural networks (CNNs) and bidirectional recurrent neural networks (BRNNs). This integration allows the model to effectively capture both local sequence motifs and long-range dependencies within protein sequences.

Moreover, DeepPredict leverages pre-trained protein language model (PLM) embeddings, specifically ESM-2 (Lin et al., 2023), to achieve high predictive accuracy while maintaining computational efficiency. Unlike traditional methods that rely on multiple sequence alignments (MSAs), DeepPredict eliminates the need for MSAs, significantly reducing computational overhead while preserving predictive performance.

DeepPredict consists of two primary prediction models. Porter6 is used for secondary structure prediction and employs a hybrid CBRNN architecture that efficiently captures both local sequence dependencies and long-range interactions, enabling predictions in both three-state (SS3) and eight-state (SS8) classifications. PaleAle6 is responsible for RSA prediction and uses a deep learning approach to predict RSA in three formats: binary classification (RSA_2C), four-class classification (RSA_4C), and continuous real-valued RSA predictions.

The integration of ESM-2 embeddings significantly enhances DeepPredict’s ability to infer secondary structure and solvent accessibility without relying on traditional MSAs. These embeddings are derived from transformer-based protein language models trained on large-scale protein sequence databases, allowing the model to capture both local and global sequence dependencies. By leveraging ESM-2, DeepPredict improves predictive performance for both Porter6 and PaleAle6 while eliminating the computational costs associated with evolutionary profile-based methods such as Porter5 (Torrisi et al., 2019) and PaleAle5 (Kaleel et al., 2019).

DeepPredict’s model architecture efficiently processes protein sequences using CBRNN layers and ESM-2 embeddings. Due to limitations of the ESM-2 model, DeepPredict supports sequences up to 1,022 residues. The convolutional layers capture local sequence dependencies, while the bidirectional recurrent layers extract long-range interactions, ensuring accurate secondary structure and RSA predictions within the supported sequence length range. Despite the depth of the model, DeepPredict remains computationally efficient, balancing high predictive accuracy with minimal resource consumption.

By integrating these advanced deep learning architectures and embedding techniques, DeepPredict provides a fast, scalable, and reliable solution for protein secondary structure and solvent accessibility predictions. This makes it a valuable tool for researchers in structural bioinformatics, offering an efficient alternative to traditional MSA-dependent methods.

### 2.2 Data sources and benchmarking

DeepPredict was trained and validated using high-quality datasets derived from experimentally resolved protein structures available in the Protein Data Bank (PDB) (Burley et al., 2019). These datasets were curated to ensure diversity and minimize redundancy, allowing the model to generalize effectively across different protein families. To construct a robust dataset, sequences were clustered at 30% and 80% sequence identity thresholds, ensuring that the training and test sets remained non-redundant.

For secondary structure prediction, Porter6 was trained on experimentally annotated secondary structure assignments derived from the DSSP (Cuff and Barton, 1999) (Define Secondary Structure of Proteins) program. DSSP assigns secondary structure elements based on hydrogen bonding patterns in high-resolution crystal structures. Protein secondary structure prediction (PSSP) is typically divided into three-state (SS3) and eight-state (SS8) classifications, with the latter providing a more detailed description of structural elements, including additional motifs such as the 310-helix (G), isolated β-bridge (B), bend (S), turn (T), and π-helix (I). Secondary structure is mapped from the 8 DSSP classes into three classes as follows: H, G, I → Helix; E, B → Strand; S, T,. → Coil.

For relative solvent accessibility (RSA) prediction, PaleAle6 was trained on solvent accessibility values computed from DSSP-derived absolute solvent accessibility (ASA) values. These values were normalized using the standard formula (Equation 1):

$$RSA_{i}=\frac{SA_{i}}{MAX_{i}}\;*\;100\%$$ (1)

where *SAi* is the solvent accessibility of residue *i* (in Å^2^) from DSSP, and *MAXi* is the maximum solvent accessibility for that amino acid *i* (in Å^2^) type [19]. For classification, amino acids were grouped into four RSA classes: [0%–3%], [4%–24%], [25%–49%], and [50– ∞ %], chosen to maintain balanced class distributions. For binary classification, the ranges [0%–24%] and [25– ∞ %] were used.

To rigorously assess DeepPredict, the model was evaluated on the 2022 Test Set (Alanazi et al., 2025a) and an independent 2024 Test Set (Alanazi et al., 2025a), and compared with other state-of-the-art prediction tools.

• 2022 Test Set: Consisting of 5,130 non-redundant protein sequences, this dataset was curated using a 30% sequence identity threshold to minimize homology bias and ensure diversity.
• 2024 Test Set: An independent benchmark dataset containing 692 newly released PDB entries, clustered at 30% sequence identity against the training set, ensuring an unbiased evaluation of the model’s generalization ability.

This benchmarking framework ensures that DeepPredict maintains high predictive accuracy and robustness across different protein families, demonstrating its effectiveness in protein secondary structure and solvent accessibility prediction.

### 2.3 Performance

DeepPredict was rigorously validated using the 2022 test set, which consists of 5,130 non-redundant protein sequences clustered at 30% sequence identity. The model was further evaluated on the 2024 test set, an independent dataset of 692 newly released PDB sequences, ensuring a fair assessment of generalization. The evaluation metrics included:

• Accuracy (ACC) for secondary structure prediction (Q3 and Q8).
• Pearson Correlation Coefficient (PCC) for real-valued RSA prediction.
• Accuracy (ACC) for RSA_2C and RSA_4C classifications.

#### 2.3.1 Performance on the 2022 test set

DeepPredict achieved high accuracy across all prediction tasks, demonstrating state-of-the-art performance, as shown in Table 1. It successfully balances computational efficiency and predictive power by eliminating the need for multiple sequence alignments (MSAs) while maintaining high predictive accuracy.

**TABLE 1:** Results of 2022 test set validation.

Prediction task	Metric	DeepPredict performance (2022 test set)
Secondary Structure (SS3)	Accuracy (ACC)	86.34%
Secondary Structure (SS8)	Accuracy (ACC)	75.23%
Binary RSA (RSA_2C)	Accuracy (ACC)	82.48%
Four-Class RSA (RSA_4C)	Accuracy (ACC)	59.60%
Real-Valued RSA	Pearson’s (PCC)	77.88

#### 2.3.2 Comparison with other state-of-the-art predictors (2024 test set)

The performance of DeepPredict was compared against state-of-the-art secondary structure and RSA predictors, including NetSurfP-3.0 (Høie et al., 2022), NetSurfP-2.0 (Klausen et al., 2019), SPOT-1D-LM (Singh et al., 2022), Porter5 (Torrisi et al., 2019), and PaleAle5 (Kaleel et al., 2019)models, as summarized in Table 2. DeepPredict demonstrated competitive performance, outperforming previous predictors in RSA prediction while maintaining high accuracy in secondary structure prediction. By leveraging ESM-2 embeddings instead of traditional MSA-based approaches, DeepPredict achieves high efficiency without sacrificing predictive power, making it an optimal tool for large-scale and single-sequence protein structural analyses.

**TABLE 2:** Performance comparison with other predictors on the 2024 test set.

Method	SS3 (Q3)	SS8 (Q8)	RSA_2C ACC	RSA_2C F1	RSA_4C ACC	RSA_4C F1	Real-value RSA PCC
DeepPredict (Porter6 & PaleAle6)	84.56%	74.18%	79.74%	0.79	55.30%	0.54	73.08%
NetSurfP-3.0	82.92%	71.84%	77.35%	0.78	—	—	69.22%
NetSurfP-2.0	81.37%	70.06%	77.17%	0.78	—	—	68.60%
Porter5	81.03%	70.03%		—	—	—	—
PaleAle 5.0	—	—	77.01%	0.76	51.54%	0.51	—
SPOT-1D-LM	84.30%	74.09%	78.34%	0.79	—	—	70.50%

DeepPredict (Porter6 and PaleAle6) consistently outperformed existing methods across all tasks.

## 3 Web server description

DeepPredict is a user-friendly web server designed for real-time protein structure prediction, targeted at both bioinformatics and structural biology researchers. The interface allows users to submit sequences in FASTA format and receive structured results with confidence scores.

### 3.1 Web interface overview

**FIGURE 1:**
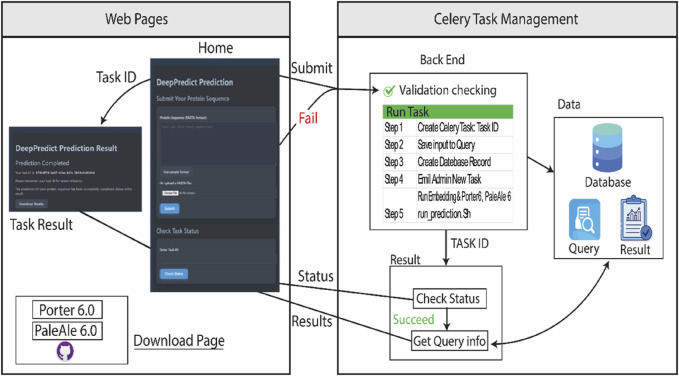
DeepPredict Web Server Framework. The diagram depicts the structural layout of the DeepPredict web server. The left section represents the primary web pages: (1) the Home Page, which allows users to submit tasks, check status updates, access general information, and download links; (2) the Task Result Page, where users can view task-specific details and download outputs; and (3) the Download Page, which provides installation guidelines for local deployment. The right section illustrates the back-end architecture, highlighting key components such as page interactions, Celery-based task management, data processing, and the execution of prediction tasks.

The website framework includes three primary web pages to facilitate user interaction and access to DeepPredict web server’s functionalities (web pages section, left part in Figure 1):

• Home Page: This serves as the primary interface, offering users multiple options to submit protein sequences in FASTA format, track the status of submitted tasks, and access essential information about DeepPredict. Additionally, it provides a direct link to the Download Page, allowing users to explore local installation options.
• Task Result Page: Once a sequence has been submitted, this page provides a unique task identifier and displays the current status of the process, indicating whether the prediction is pending or completed. Users can download the final results upon task completion.
• Download Page: This section provides comprehensive installation instructions for those who wish to run DeepPredict (Porter6 & PaleAle6) locally. It outlines the installation method and directs users to the software repository on GitHub.

The web server is designed to be intuitive and efficient, ensuring a smooth user experience while enabling fast and accurate protein structure predictions with minimal computational complexity.

### 3.2 Back-end implementation

The DeepPredict web server is developed using the Django (Django Software Foundation, 2024) framework, with Python managing the back-end operations and HTML and JavaScript supporting the front-end interface. User requests are processed asynchronously, with each request handled as an independent task. To facilitate this, Celery (Solem, 2023) is integrated, allowing prediction tasks to be executed separately from the main application thread. This asynchronous architecture enables users to check their task status later using a unique task ID, eliminating the need to keep the page open during processing.

Prediction results are retained for 1 week, giving users sufficient time to download and analyse their data. Since DeepPredict does not depend on MSAs and operates with ESM-2 embeddings, which require minimal computational resources, the overall system load is kept low, processing approximately 50 sequences per batch. Tasks are managed in a first-come, first-served queue within Celery, ensuring an efficient workflow without overloading system resources. This approach minimizes wait times while maintaining fast prediction speeds.

To efficiently manage data, DeepPredict employs a hybrid storage solution (Figure 1, right side), comprising:

• Relational Database (PostgreSQL) (PostgreSQL GlobalDevelopment Group, 2023): Stores essential metadata for each task, including task ID, timestamps, file paths to input/output files, and other relevant details. By restricting database storage to metadata, server performance is optimized, preventing unnecessary load.
• File System Storage: Large files, such as sequence data and prediction results, are stored directly on the server’s disk. This prevents database congestion, ensuring users can efficiently download large result files without impacting overall database performance.

This architecture ensures that the web server can handle multiple concurrent users and large datasets efficiently, while keeping computational and operational costs low.

### 3.3 Implementation and run time

DeepPredict is designed for efficient deep learning inference, utilizing asynchronous task management via Celery to ensure smooth processing of user requests, even under heavy traffic.

The system runs on a Linux-based environment, powered by a 24-core CPU, 256 GB DDR5 RAM, and an NVIDIA RTX 4000 ADA GPU (20 GB VRAM). Storage includes 6 TB across high-speed NVMe and SATA SSDs, with a RAID 1 configuration for data redundancy and reliability.

With GPU acceleration, high-speed SSDs, and optimized multi-core processing, DeepPredict efficiently handles large-scale sequence analyses, batch processing, and real-time predictions. DeepPredict also supports input files up to 40MB, making it well-suited for high-throughput applications.

Using the UniProt Reviewed Swiss-Prot15 human proteome dataset (The UniProt Consortium et al., 2023), which originally contained 20,421 sequences and was reduced to 18,115 by excluding sequences longer than 1,022 residues (totaling 8.8 MB), our server successfully completed the predictions in 9,938.89 s, achieving an average runtime of 0.55 s per sequence. When estimating prediction time for a 1,000-residue sequence, our method yields an average of 1.33 s, demonstrating efficiency for large-scale and high-throughput applications.

### 3.4 User guide

As shown in Figure 1, users can submit a query from the homepage by either entering one or more protein sequences directly into the text area or uploading a FASTA file (.fasta). Regardless of the submission method, the input must be formatted in FASTA format.

For example, submitting a file named p49913.fasta, containing a UniProt sequence (P68871), will initiate a prediction task. Once the query is submitted, a task is created immediately, and the user is redirected to the Task Result Page, where a unique task ID (e.g., a853da32-9fe5-4c73-8f85-7c6324c194ac) is assigned.

**FIGURE 2:**
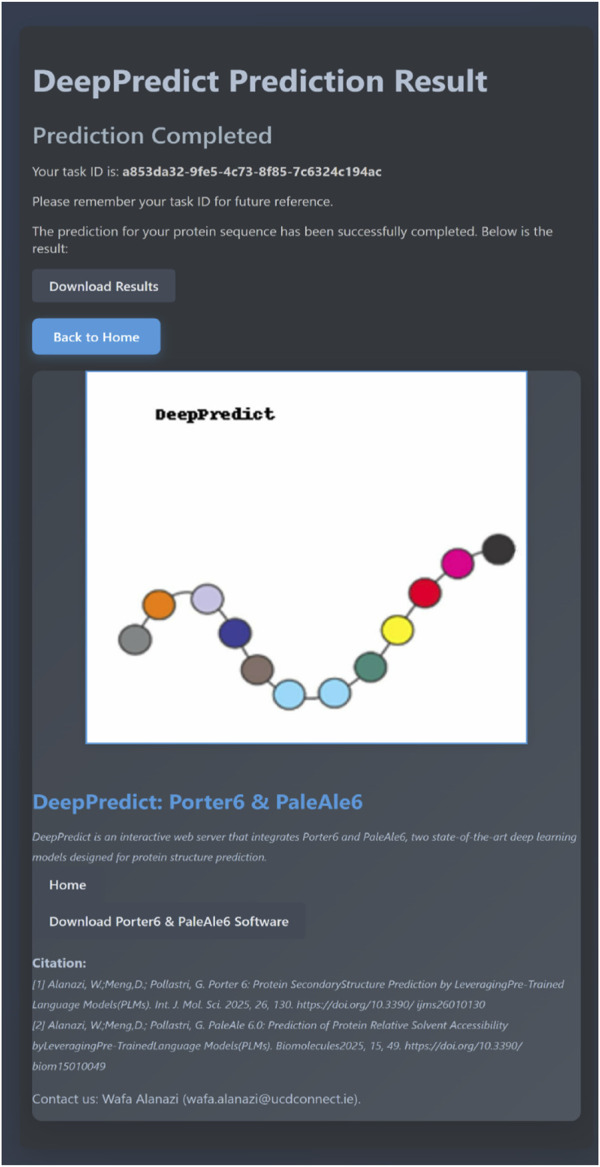
This screenshot shows the prediction result of Uniprot sequence P49913.

Users can refresh the result page or save the task ID to check the task status later from the homepage. Once the prediction is completed, a download link appears, enabling users to download the results as CSV files, with all sequences saved in a separate file for each prediction type (illustrated in Figure 2 for sequence P68871).

## 4 Limitations and Future work

While DeepPredict achieves state-of-the-art performance without relying on MSAs, certain limitations remain. The model’s performance may be affected for sequences approaching the maximum supported length of 1,022 residues, as ESM-2 embeddings are truncated beyond this point. Future versions may explore improved handling of longer sequences and additional output features to support integration into broader structural biology workflows.

## 5 Conclusion

DeepPredict integrates Porter6 for secondary structure and PaleAle6 for solvent accessibility prediction, offering a fast, accurate platform for protein structural analysis. Its high-performance architecture enables both high-throughput analyses and detailed individual investigations, delivering state-of-the-art predictions without relying on MSAs.

Leveraging pre-trained language models and deep learning, DeepPredict ensures precision and speed, making it a valuable resource for the bioinformatics community. The user-friendly interface features well-structured pages for query submission and task status monitoring, while a robust data management system securely handles large datasets, temporarily storing results for user retrieval and analysis.

With free access, clear documentation, and downloadable resources, DeepPredict serves as a versatile tool for researchers in bioinformatics and structural biology. Thanks to its scalable architecture and flexibility, DeepPredict represents a sustainable long-term solution for protein secondary structure and solvent accessibility prediction. The complete training and evaluation datasets, including PDB IDs, are freely available via the GitHub repositories, supporting transparency and reproducibility.

## Data Availability

The datasets presented in this study can be found in online repositories. Website: [DeepPredict Prediction]. GitHub Repository (datasets and code): Porter6: https://github.com/WafaAlanazi/Porter6. PaleAle6: https://github.com/WafaAlanazi/PaleAle6.
